# Influence of dental arch width changes on the effective space required to align anterior teeth

**DOI:** 10.1007/s00784-025-06361-x

**Published:** 2025-05-13

**Authors:** Fayez Elkholy, Catrin Gerhart, Falko Schmidt, Bernd G. Lapatki

**Affiliations:** Department of Orthodontics, Ulm University, Albert-Einstein-Allee 11, 89081 Ulm, Germany

**Keywords:** Modell analysis, Space requirement, Digital setup, Arch width change

## Abstract

**Aim:**

Traditionally, the widest mesio-distal tooth dimensions are used to analyze space requirements in treatment planning. However, in reality, it is the arch form dependent interproximal contact locations that determine the space required for tooth alignment. The aim of this study was to evaluate the influence of expansion and constriction of dental arch width on the space required for alignment of upper and lower incisors.

**Materials and methods:**

Fifty digital dental arch model pairs were segmented and aligned using OnyxCeph 3D™ software (Image Instruments, Germany). 3D coordinates of actual interproximal contact points were extracted from the digital setups. The mesio-distal space requirement for each tooth was determined by measuring the linear distance between its interproximal contact points projected on the occlusal plane. The dental arch was then expanded and narrowed in 2-mm increments at its distal ends, and the space requirement for each incisor was determined again after each increment.

**Results:**

Statistical analysis using linear models revealed a small increase in space required for incisor alignment with increasing arch width (p < 0.05). An average increase in space requirement of 0.03 mm and 0.04 mm was observed per 1-mm expansion of the maxillary and mandibular arches, respectively. The corresponding values ​​for constriction were 0.05 mm per 1-mm arch width change for both jaws.

**Conclusion:**

The influence of dental arch form on the mesio-distal space required for incisor alignment is negligible. Hence, this factor may be ignored in the decision to apply dental arch expansion or premolar extraction in patients with anterior crowding.

## Introduction

Crowding is one of the main types of malocclusion and the most common reason why patients seek orthodontic therapy [1, 2]. Accurate analysis of the space required for tooth alignment is crucial for a stable therapeutic result [3, 4]. Space analysis is especially important for individual planning of premolar extraction or non-extraction therapy in borderline cases. In a systematic review, Kirschen et al., [5] evaluated the different approaches for space analysis. Most studies included in this review used the widest mesio-distal width of each tooth to assess the space required for alignment [4, 6–12], but other methods were also used. For example, some studies used physical dental arch models and a caliper gauge [4, 6–10, 13] or a pair of dividers with sharpened ends [3, 14]. Others used virtual models and orthodontic analysis software such as OrthoCAD™ (Cadent, Inc, Fairview, NJ) [6] or OnyxCeph3™ (Image Instruments GmbH, Chemnitz, Deutschland) [15].

An important difference between these alternative methods for space analysis concerns the exact locations at which the widest mesio-distal tooth dimension was acquired. Keene et al. described these measurement points in the upper incisal third or even at the incisal edges [16], while Johal and Battagel took measurements at the contact points of teeth with the widest mesio-distal tooth dimensions [3]. In contrast, Hunter and Priest took measurements from the mesial and distal contact areas of each tooth in relation to the tooth axis. These authors emphasized that the widest spot is situated more buccally than the contact areas, and that this location often does not correspond to the real contact points of teeth in an aligned dental arch. However, the difference between the measurement at the contact points and the widest mesio-distal area of the tooth were insignificant for determining the space needed for alignment [17]. Lundström further measured the mesio-distal width of the tooth from the anatomical contact points projected on the occlusal plane in correct occlusion [13].

**Fig. 1:**
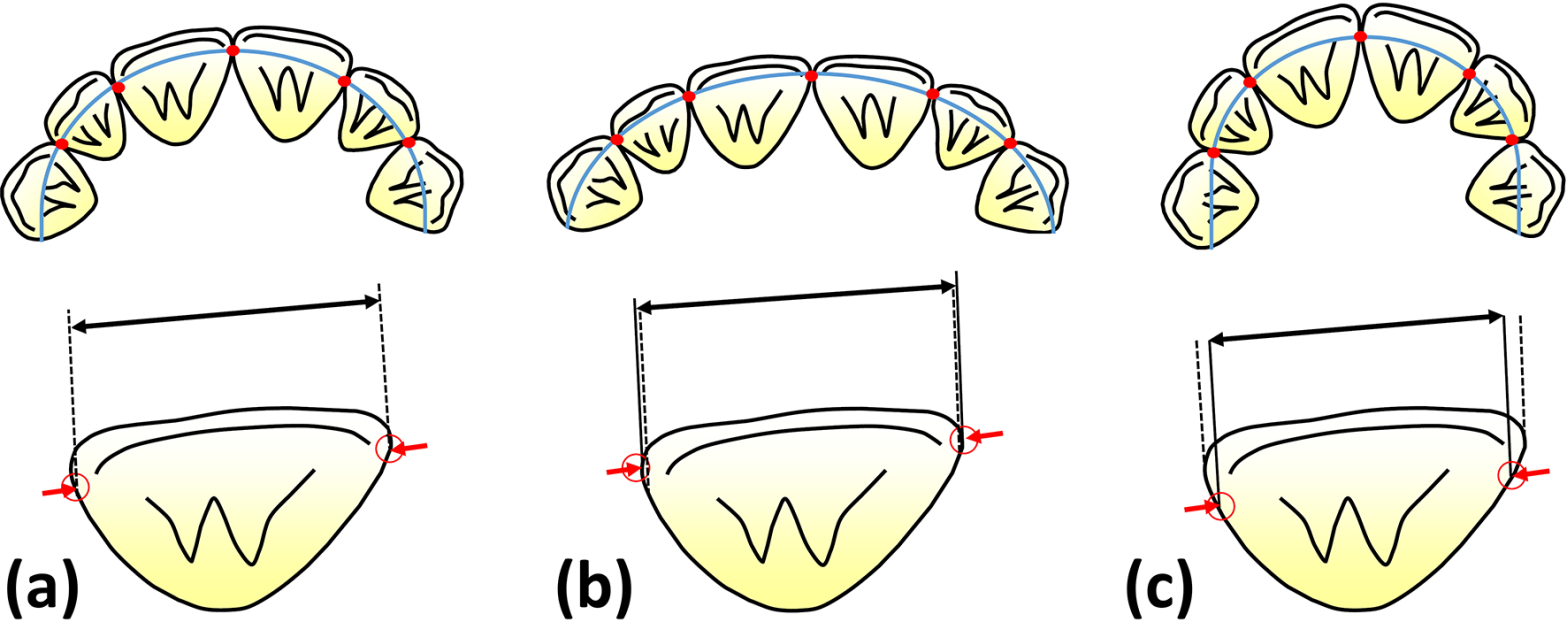
Interproximal contact point locations (tips of red arrows) and expected changes of required mesio-distal space for this tooth after alignment (black arrow). (**a**) Alignment according to the original arch form. The dotted lines represent the space required for the tooth in this situation. (**b**) Shift of interproximal contact points and resulting change of required mesio-distal space after expansion and (**c**) after constriction of the dental arch. Dotted lines of (a) are also illustrated in (b) and (c) for comparison

In patients with anterior crowding, expanding the dental arch is a common alternative to extracting premolars to gain space in the dental arch without changing the antero-posterior incisor positions and to avoid flattening the facial profile [18]. Previous studies have described different amounts of space gained by dental arch expansion [5, 18, 19, 20, 21], but none of these studies have examined the possibility that therapeutic dental arch changes may alter the amount of space actually required for anterior tooth alignment. Furthermore, studies that used dental contact points based these points on malocclusion models, and the locations of these points were considered static. Consequently, changes in contact point locations due to alignment of the anterior segment combined with either expansion or constriction of the dental arch were not considered, nor was the possible influence of this on the mesio-distal space required for incisor alignment (Fig. 1). In the current study, we evaluated these aspects by controlled dental arch expansion or constriction based on digital models of patients with crowding in the upper and lower anterior segments.

## Materials and methods

### Model selection and preparation

Digital maxillary and mandibular dental arch models documenting the pretherapeutic situation of 50 patients were evaluated. Digital models were obtained from impressions taken using an alginate material (Blueprint^®^ Xcreme; Dentsply DeTrey, Konstanz, Germany) and a mixing device (AM 501; Hauschild, Hamm, Germany). From these impressions, plaster cast models were fabricated. This method is considered an accurate standard for diagnostic purposes [22]. The selected models were than scanned using a desktop scanner (d-STATION3D, Breuckmann GmbH, Meersburg, Germany).

The inclusion criteria for this study were:

- permanent dentition or transitional dentition with fully erupted permanent incisors.
- no spacing between the teeth 6–6.
- no tooth aplasia or tooth form anomalies.
- no loss of hard tooth structure due to caries or trauma.
- no dental restorations including approximal surfaces or artificial teeth (crowns, partial crowns, or veneers).

Patients were consecutively recruited from those seeking treatment in the Department of Orthodontics, University of Ulm, Germany. Neither gender nor ethnic background were considered in the selection process. In total, 36 female and 14 male patient records were included. After recruitment, included model pairs were blinded and randomly numbered.

### Digital setup procedures

Digital model preparation and digital setup of the upper and lower dental arches were performed in the *VTO 3D* module of OnyxCeph^3TM^ (Version 3.2.45 (92), Image Instruments GmbH, Chemnitz, Germany).

### Segmentation of the full-arch model into individual tooth objects

**Fig. 2:**
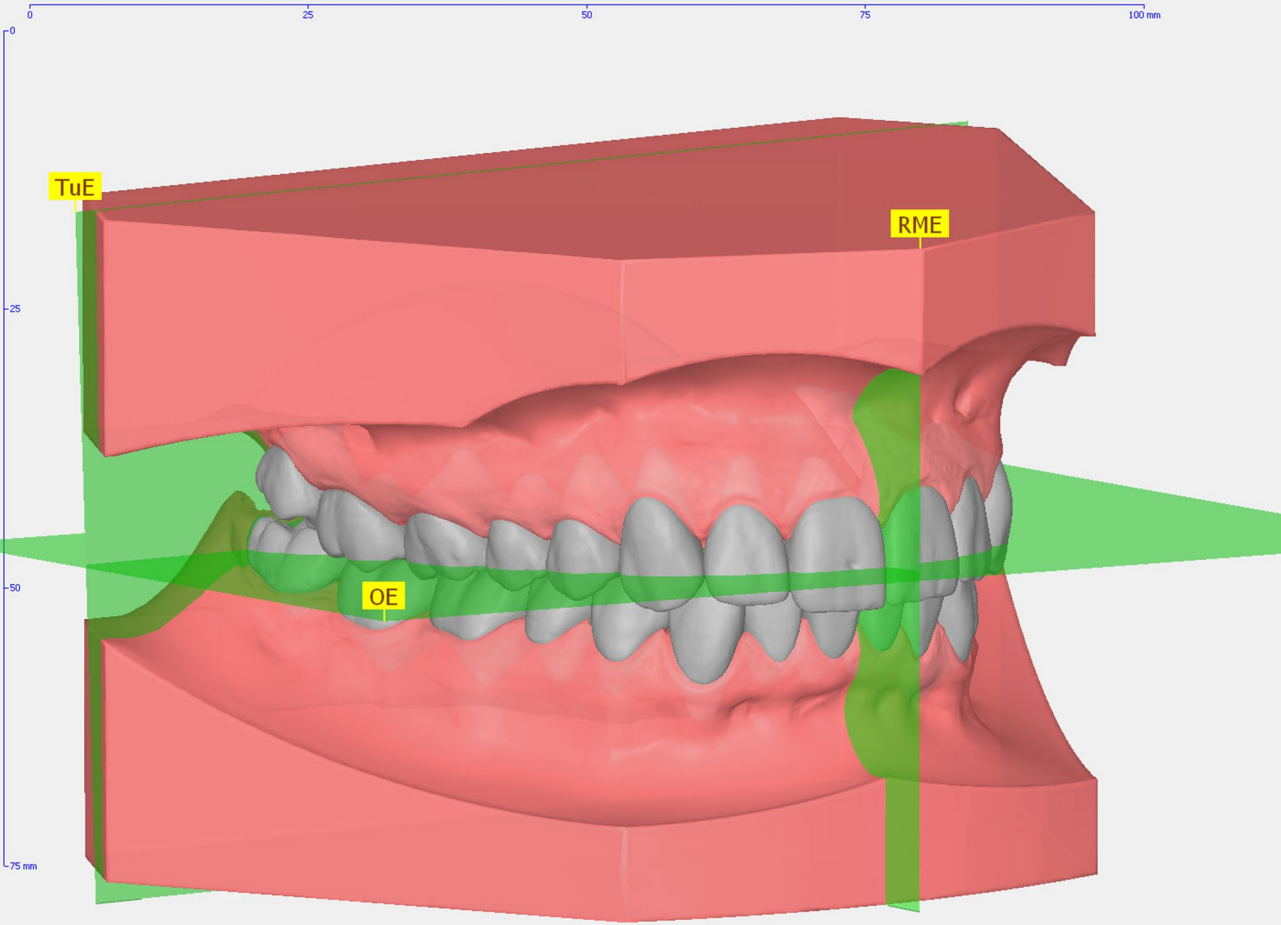
Coordinates and reference planes for an aligned and model imported in the orthodontic analysis software Onyxceph^3TM^. The different coordinates were oriented on different planes determined in Onyxceph^3TM^ including the transversal “occlusal” plane (OE), the anteroposterior plane (RME), and the vertical plane (TuE)

**Fig. 3:**
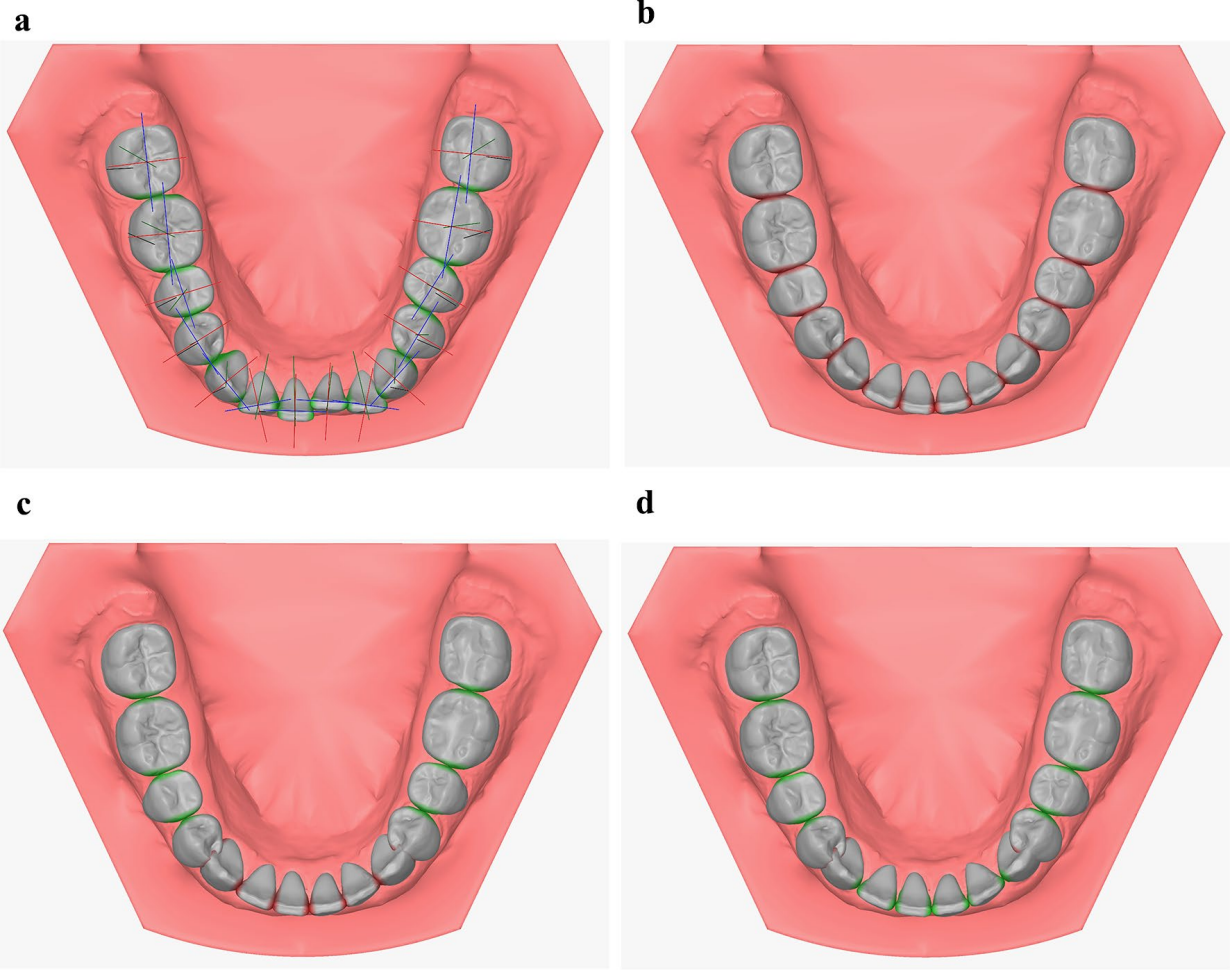
Different stages of equalizing the space discrepancy after correction of the anterior segment. Note the color changes of approximal surfaces: red for overlapping contact points and green for correct alignment. (**a**) Original model after tooth segmentation showing the different tooth coordinates in mesio-distal (blue), vestibular (red) and vertical (green) directions. (**b**) Model after tooth alignment showing the crown overlapping (red regions). (**c**) Model after discrepancy equalization in the posterior segment (green regions indicate a direct contact without overlapping). (**d**) Model after discrepancy equalization and positioning of the canines in mesio-distal direction in OnyxCeph3TM

First, a jaw coordinate system was defined according to the occlusal and mid-palatal plane of the occluded model pair (Fig. 2). Then, maxillary and mandibular dental arches were segmented to create a single 3D object of each individual tooth with its own coordinate system (Fig. 3a). The points on the crown surfaces defining the widest mesio-distal crown dimensions (i.e., the mesial and distal crown points) were automatically determined by the software based on automatic selection of a model crown with predetermined mesial and distal crown points, LA point, and individual tooth coordinate system from its integrated tooth data base. This coordinate system was defined as follows: the mesio-distal axis was defined by the widest mesio-distal points of the crown, the vertical tooth axis as the incisal point and the estimated root apex, and the vestibular axis as perpendicular to the mesio-distal and tooth axis.

### Digital setup of dental arches

As a preliminary step, the individual dental arch form was defined according to the mathematical description by Bezier [23]. The anterior fix point of this arch was the midpoint between the mesial crown points of both central incisors, and the posterior fix points were the most distal points of the crowns of the most distal right and left molars. The width of the Bezier curve was adapted to obtain a best fit curve passing through or close to the mesial and distal crown points of all individual teeth of the unaligned arch. If applicable, the anterior fix point was corrected to achieve dental arch symmetry in the transversal and sagittal dimensions.

The dental arch was set up by one examiner as follows:

- All teeth were aligned by positioning their mesial and distal crown points on the individual dental arch. The three vectors of the 3D coordinate systems describing the translations of individual teeth in this alignment were parallel to the tangent on the dental arch curve. This alignment resulted in overlapping of the approximal tooth surfaces which is indicated by their red color (Fig. 3b).
- Crown angulations and inclinations were adjusted according to the prescription chart established by Andrews [24].
- The space discrepancy in each quadrant was equalized by eliminating the approximal tooth surface overlaps with an accuracy of 0.01 mm at both posterior segments by sequential mesialization of the first molar, second premolar, and first premolar turning the approximal surfaces green (Fig. 3c). The same procedure was then performed for the anterior segment beginning at the dental midline in the order central incisors, lateral incisors, and canines. This resulted in an overlap between the canines and first premolars (red marking) in each quadrant because the distal crown points of the most distal molars were kept in their original location (Fig. 3d).

### Determination of initial space required for alignment of the incisors

The space required for incisor alignment was determined using the CAD software CATIA (Dassault Systèmes, Vélizy Villacoublay, France). For this purpose, the mesial and distal contact points of each aligned incisor were projected on the occlusal plane and the linear distance between the two projected contact points (i.e., the effective mesio-distal space of the corresponding incisor) was determined. The total space required for alignment of the entire incisor segment was calculated by summing up the effective mesio-distal space requirements of all four incisors.

### Setups with expanded or constricted dental arches

**Fig. 4:**
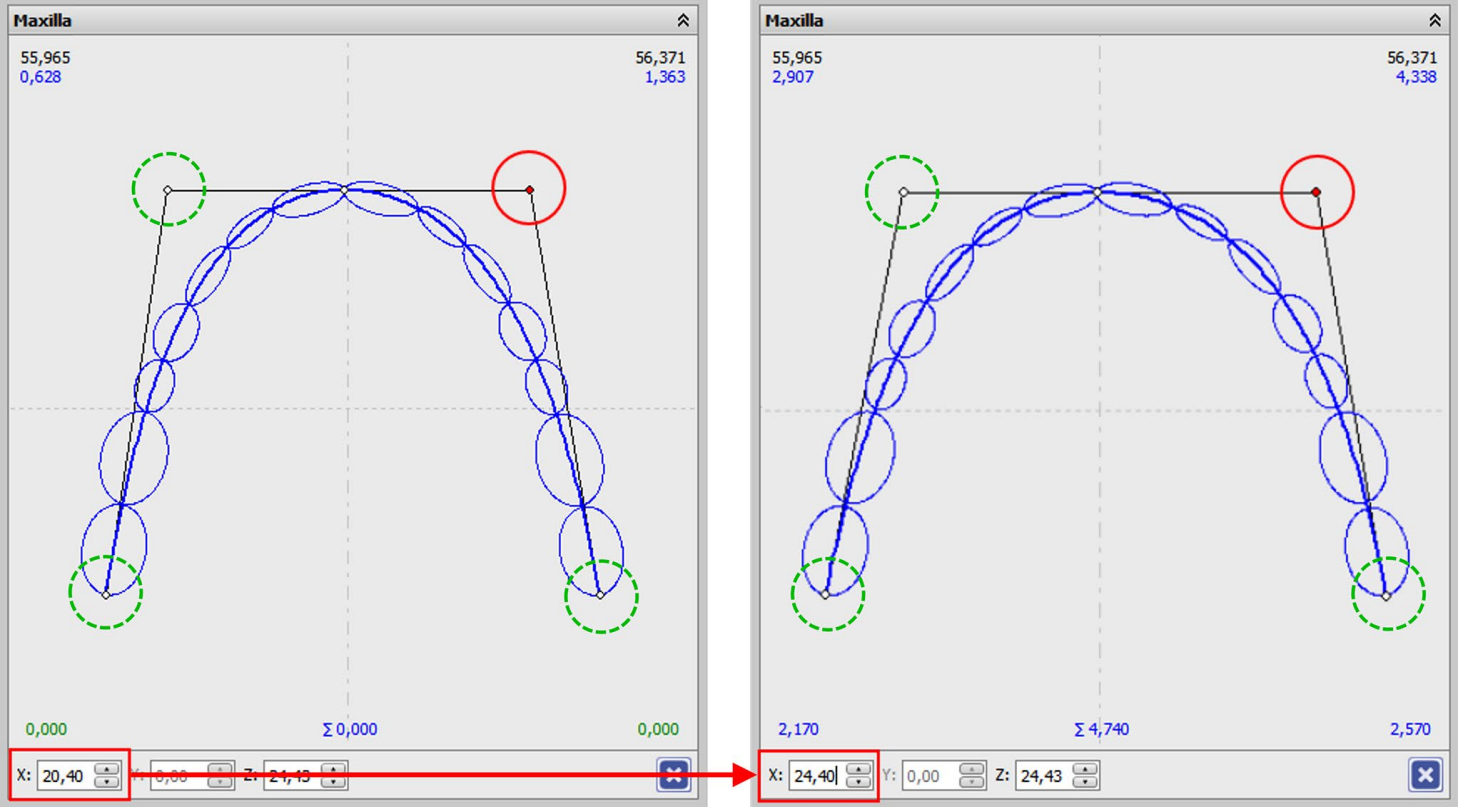
Screenshot of the arch width change. The circled points represent the knot points for the arch width change. The points were moved in the x-axis (i.e., horizontally for 1 mm in each step). The example shows the expansion at the red marked point on 4 mm. Applying this to the opposite point achieves an arch expansion of 8 mm

Additional setups with modifications of dental arch width comprised stepwise expansions of up to 8 mm and constrictions of up to 6 mm in 2-mm steps each, while maintaining a Bezier curve as dental arch form. These expansion or constriction values were applied to the posterior ends of the dental arch (i.e., the most distal molar points) as well as the anterior points on the dental arch tangent (Fig. 4). Corresponding changes in intercanine distances were on average 62% (range 54–71%) smaller than the changes in intermolar distances. The effective excess or deficit in space was then determined after each expansion or constriction step as described above. As a result, each modification in dental arch width gave the distinct mesio-distal spaces required for effective incisor alignment.

### Data analysis

All statistical analyses were conducted in R (R Foundation for Statistical Computing, Vienna, Austria) and included calculation of median and interquartile ranges for the different spaces required for individual incisors and upper and lower incisor segments after dental arch width was modified. These variables were also determined for the corresponding 3D displacements of the interproximal contact locations.

A linear model was used to characterize the interrelation between the changes in dental arch width and the required or excess space after dental arch width was modified. The calculation also estimated the rate of change of the effective space required for both expansion and constriction of the maxillary and mandibular dental arches.

## Results

### Absolute effective mesio-distal widths of the incisor segments and of individual incisors

**Fig. 5:**
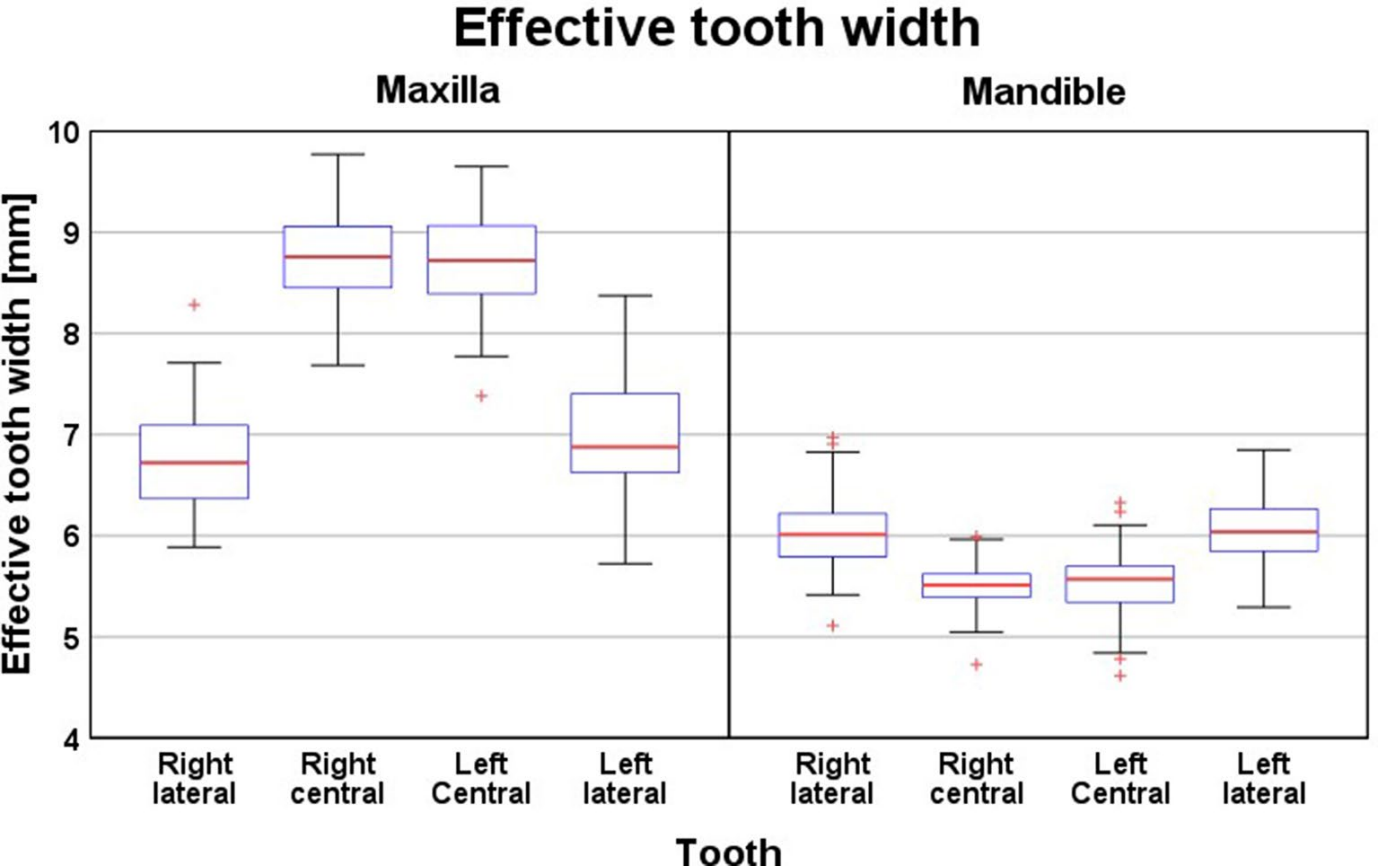
Effective maxillary and mandibular incisor widths measured at the contact points of each tooth on the original models before changing the arch width

Figure 5 illustrates the effective mesio-distal widths of the individual maxillary and mandibular incisors determined after alignment according to the original arch form. In general, a symmetry was observed between the central and lateral incisors in each jaw. The upper central and lateral incisors showed a relation of 1:0.78. The mandibular incisors showed an opposite relation with the lateral incisors circa 1.09 times wider than the central incisors (mean difference, 0.48 mm). The grand median for the total effective width was 31.10 mm for the upper incisors and 22.98 mm for the lower incisors. The relation between the total width of the upper and lower incisors nearly coincided with the relation described by Tonn [25] with a mean difference of only 0.13 mm. The large interquartile range of 7.03 mm, however, indicates high variability of the difference between these relations.

### Arch width-related changes in effective mesio-distal width of the incisor segments

**Fig. 6:**
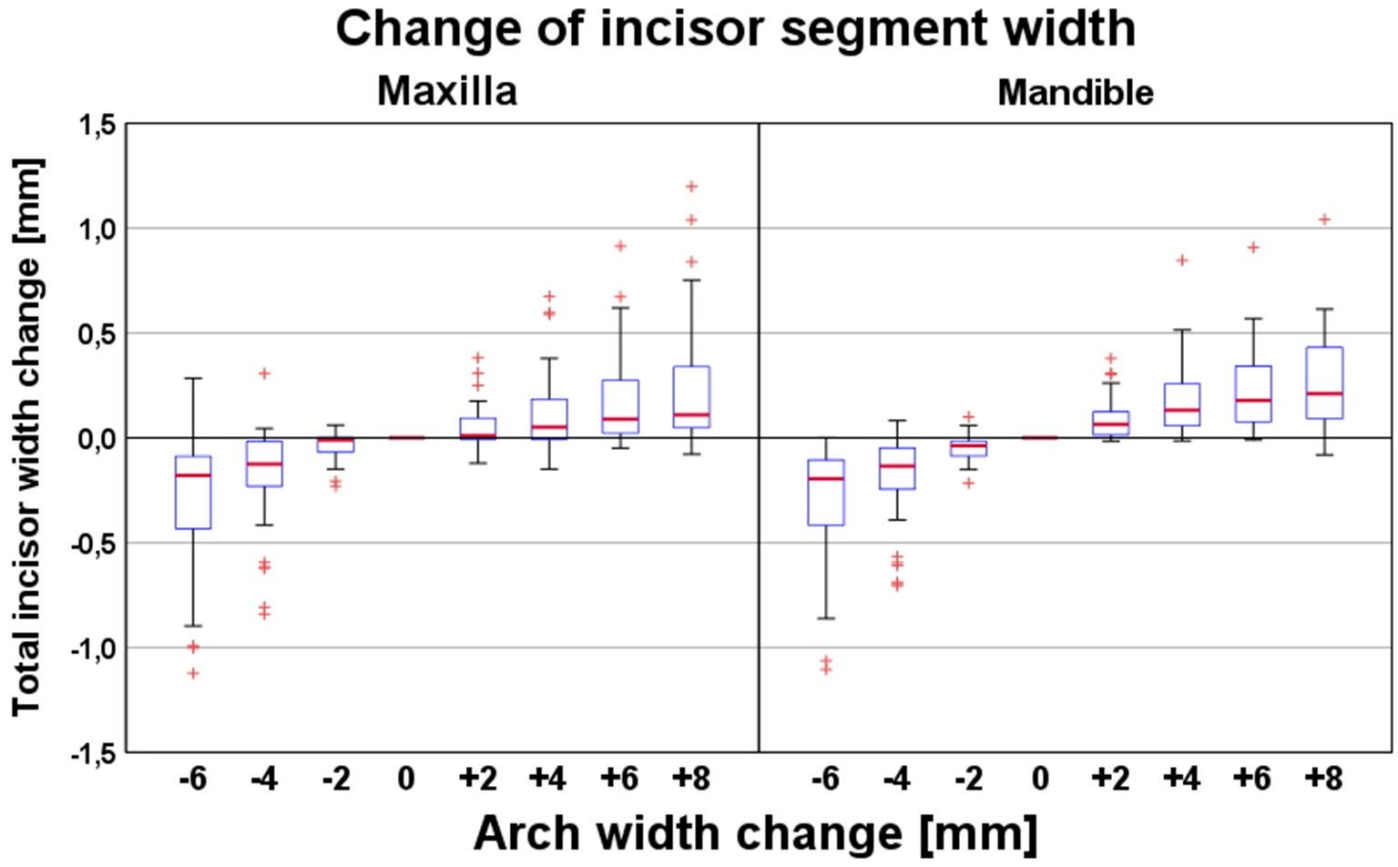
Changes of effective mesio-distal width of the maxillary (left panel) and mandibular incisor segment (right panel) depending on the amount of expansion (+) or constriction (-) of the dental arch. The latter variable (expansion/constriction) refers to the most distal points of the second molar crowns

As illustrated in Fig. 6, the effective mesio-distal space required for alignment of the four maxillary or mandibular incisors correlated positively with dental arch width. However, this effect was relatively small (Table 1). For instance, a 6-mm constriction of the dental arch decreased the effective mesio-distal width of the incisor segments by − 0.18 mm (maxilla) and − 0.20 (mandible), whereas a 6-mm expansion increased the mesio-distal incisor width by 0.11 mm (maxilla) and 0.21 mm (mandible).

**Table 1:** Changes in effective mesio-distal width of individual maxillary and mandibular incisors depending on the amount of dental arch expansion (+) or constriction (-). Arch width changes were measured at the most distal points of the Bezier curve of the dental arch. The values refer to the mesio-distal width of the incisors with the original arch width (central column). The values represent the median values and interquartile ranges are given in brackets

Jaw	Tooth	Dental arch compression			Original	Dental arch expansion			
		−6	−4	−2	Total eff. tooth width (mm)	+ 2	+ 4	+ 6	+ 8
Maxilla	Incisor segment	-	-	-	31.10 (6.75)	-	-	-	-
	Right lateral	0.00 (0.80)	0.00 (0.78)	0.00 (0.26)	6.72 (2.40)	0.00 (0.54)	0.00 (0.59)	0.00 (0.61)	0.00 (0.69)
	Right central	−0.05 (0.99)	−0.04 (0.77)	0.00 (0.67)	8.76 (2.09)	0.00 (0.48)	0.01 (0.56)	0.01 (0.54)	0.02 (0.61)
	Left central	−0.07 (0.79)	−0.02 (0.52)	0.00 (0.37)	8.72 (2.27)	0.00 (0.46)	0.01 (0.47)	0.02 (0.53)	0.03 (0.59)
	Left lateral	−0.01 (0.57)	0.00 (0.36)	0.00 (0.14)	6.88 (2.65)	0.00 (0.48)	0.00 (0.48)	0.01 (0.63)	0.01 (0.61)
Mandible	Incisor segment	-	-	-	22.98 (5.96)	-	-	-	-
	Right lateral	−0.02 (0.35)	−0.02 (0.35)	−0.01 (0.32)	6.01 (1.86)	0.01 (0.39)	0.03 (0.40)	0.03 (0.38)	0.04 (0.38)
	Right central	−0.07 (0.79)	−0.03 (0.60)	−0.01 (0.49)	5.51 (1.66)	0.00 (0.44)	0.01 (0.47)	0.03 (0.46)	0.04 (0.47)
	Left central	−0.06 (0.85)	−0.03 (0.80)	−0.01 (0.68)	5.57 (1.71)	0.01 (0.53)	0.02 (0.57)	0.04 (0.57)	0.04 (0.57)
	Left lateral	−0.01 (0.88)	−0.02 (0.43)	−0.01 (0.34)	6.04 (1.56)	0.01 (0.70)	0.02 (0.71)	0.04 (0.71)	0.05 (0.76)

As estimated by the linear model (Table 2), each 1-mm constriction of the dental arch increased the effective mesio-distal width of the incisor segment by 0.05 mm in both the maxillary and mandibular arches (*P* < 0.01). In comparison, the effective mesio-distal space required increased only by 0.03 mm (maxilla) and 0.04 mm (mandible) per 1-mm arch expansion (*P* < 0.01).

**Table 2:** Statistical estimates of the changes in effective mesio-distal width per 1-mm expansion or constriction of the dental arch. Estimates of the 95% confidence intervals are given in brackets

Jaw	Segment/tooth	Direction of arch width change	Estimated change per mm arch width change	*P* value
Maxilla	Anterior segment 2–2	Constriction	0.05 (0.04 − 0.06)	0.000
		Expansion	0.03 (0.02 − 0.04)	0.000
	12	Constriction	0.01 (0.00 − 0.01)	0.001
		Expansion	0.01 (0.00 − 0.01)	0.000
	11	Constriction	0.02 (0.01 − 0.02)	0.000
		Expansion	0.01 (0.01 − 0.01)	0.000
	21	Constriction	0.02 (0.01 − 0.02)	0.000
		Expansion	0.01 (0.01 − 0.01)	0.000
	22	Constriction	0.01 (0.01 − 0.01)	0.000
		Expansion	0.01 (0.00 − 0.01)	0.000
Mandible	Anterior segment 2–2	Constriction	0.05 (0.04 − 0.05)	0.000
		Expansion	0.04 (0.03 − 0.04)	0.000
	42	Constriction	0.01 (0.00 − 0.01)	0.000
		Expansion	0.01 (0.01 − 0.01)	0.000
	41	Constriction	0.02 (0.02 − 0.03)	0.000
		Expansion	0.01 (0.01 − 0.01)	0.000
	31	Constriction	0.02 (0.01 − 0.02)	0.000
		Expansion	0.01 (0.01 − 0.01)	0.000
	32	Constriction	0.00 (0.00 − 0.01)	0.051
		Expansion	0.01 (0.01 − 0.01)	0.000

### Arch width-related changes in effective mesio-distal width of individual incisors

Changes in the effective mesio-distal width of the maxillary incisor segment were primarily related to the central incisors, showing maximum changes of − 0.07 mm for a 6-mm dental arch constriction and + 0.03 mm for an 8-mm expansion (Table 1). In contrast, corresponding maximum changes for maxillary lateral incisors were only ± 0.01 mm. In the mandible, changes in effective mesio-distal width with changing dental arch width were comparable between central and lateral incisors. An exception was the relatively large change in effective mesio-distal width of the lower central incisors (0.06 mm and 0.07 mm) for an extreme arch constriction of − 6 mm. The estimated changes in effective mesio-distal width of individual incisors were also statistically significant (*P* < 0.01), except for tooth 32 following constriction of the dental arch width (Table 2).

## Discussion

Previous studies have concentrated on different ways of measuring the space needed to align the frontal segments as well as the constant mesio-distal space required to align the incisors. These studies have not considered the effects of therapeutic changes in dental arch width [4, 6–10, 13], possibly because of limitations in realistic and efficient treatment simulation. In the current study, we evaluated the change in actual anterior space required by maxillary and mandibular incisors based on setups of virtual dental arch models. Orthodontic model analysis software allowed us to exactly localize the interproximal contact points, which was the basis for determining the effective mesio-distal width of individual incisors after alignment, and for simulating corresponding changes of dental arch width.

With regard to the question whether our quantitative results are clinically relevant or not we consider values > 0.5 mm as appropriate which is in accordance with previous studies [26–28]. The maximum median changes in effective mesio-distal space required by the entire maxillary and mandibular incisor segments was 0.02 mm for a 6-mm dental arch constriction and expansion. This change was clinically negligible, despite being statistically significant. We believe our conclusion is justified, even though our simulated arch width changes referred to the transversal intermolar distance, which is around 1.6 times greater than the intercanine distance. This means that the effective mesio-distal width of the incisors is nearly constant, independent of the chosen therapeutic strategy (i.e., non-extraction and dental arch expansion vs. premolar extraction and dental arch constriction). We hypothesize that the relatively small effect of dental arch expansion on the effective mesio-distal crown width is related to the relatively flat contour of the approximal crown surfaces of both maxillary and mandibular incisors. It seems that this specific incisor crown geometry keeps the effective mesio-distal width fairly stable.

An interesting finding of this study was that the difference in effective mesio-distal space required for incisor alignment was half of that for expansion of the maxillary arch compared with expansion of the mandibular arch and constriction of both maxillary or mandibular arches. Furthermore, changes in effective mesio-distal space required for alignment in the maxilla were primarily related to changes in effective mesio-distal widths of central incisors, while the effective widths of upper lateral incisors were almost stable. We hypothesize that upper lateral incisor widths were stable because of the relative palatal position of the upper lateral incisor compared with the upper central incisor, which makes the approximal contact at the lateral incisor closer to the widest mesio-distal crown diameter – i.e., at a flatter region of the interproximal crown surface. Conversely, the location of the distal approximal contact of the upper central incisors in a more palatal (i.e., more curved) crown region may explain the relatively large effect of arch expansion on the effective mesio-distal width of these teeth.

The influence of arch width modifications on the space required to align the frontal segments has already been discussed by Kirschen et al. [5]. They recommended an additional space of 0.5 mm in the entire dental arch per 1-mm expansion in the intermolar region [5]. In another study, Adkins et al. [18] investigated the space gained by arch expansion and found an increase in arch perimeter of 70% of the posterior expansion. Germane et al. demonstrated additional arch perimeters of 1.04 mm and 1.13 mm for 1-mm and 2-mm expansions, respectively [20], while Ricketts et al. found that a 1-mm expansion of the intercanine distance increased the arch perimeter by 1 mm, and a 1-mm expansion of the intermolar region increased the arch perimeter by only 0.25 mm [29]. In agreement with the results of Ricketts et al., Motoyoshi et al. demonstrated an increase in mandibular arch perimeter of 0.37 mm per 1-mm intermolar expansion in a 3D simulation study [30]. However, none of these studies considered different positions and rotations of the teeth when changing arch width, so the change in effective space needed for alignment of individual teeth was not evaluated. In comparison with these previous studies, the changes we observed in effective mesio-distal space required for alignment were very small. However, we only included the four incisors in our quantitative evaluation, so it cannot be excluded that modifying the dental arch also has a negligible effect when all frontal and buccal teeth are taken into account. This should be evaluated in further studies.

The fact that the mesio-distal space required for incisor alignment is fairly independent from dental arch form should not be interpreted as an argument against the value of digital setups for treatment planning in patients with crowding. Conventional methods usually assess the additional space required for tooth alignment based on the amount of overlap between adjacent teeth; this approach is highly sensitive to the exact antero-posterior dental arch position to which the clinician refers these quantitative assessments. Moreover, a digital setup allows not only the determination of the space discrepancy but also the simulation of changes in tooth positions and dental arch form required to eliminate crowding.

When considering measurements made on digital models, certain sources of error should be taken into consideration. These include both the digitization process and the setup generation. In this context, it may be speculated that the few outliers for effective mesio-distal widths and contact displacements shown in the boxplot diagrams may result from incomplete acquisition of approximal surfaces. This emphasizes the imperfections of optical methods for 3D scanning of dental models. This is especially true for the acquisition of interproximal tooth surfaces, which are difficult to access with optical scanners [31, 32]. The type of orthodontic treatment simulation software might also influence the changes in effective mesio-distal tooth widths reported in this study, particularly those due to differences in the implemented method for tooth segmentation and in the arch form geometries. However, studies have shown that digital models, despite minor limitations, can be more reliable than plaster models due to reduced operator variability and improved reproducibility. In particular, digital scanners often yield consistent mesio-distal measurements with clinically insignificant errors (< 0.2 mm). Therefore, while not perfect, digital reconstructions of contact points offer a valid and potentially more consistent alternative to manual plaster-based measurements [27, 33, 34, 35].

## Conclusion

- Modifications of dental arch width have a negligible influence on the effective mesio-distal space required for aligning incisor segments.
- The high stability of effective mesio-distal incisor widths may be explained by the relatively flat contour of the approximal crown surfaces of maxillary and mandibular incisors. This morphological characteristic dictates that minor alterations in the labio-lingual position of interproximal contact areas exert a negligible influence on the space required to accommodate these teeth within the dental arch.

## Data Availability

No datasets were generated or analysed during the current study.
